# The effects of cyberbullying victimization on depression and suicidal ideation among adolescents and young adults: a three year cohort study from India

**DOI:** 10.1186/s12888-022-04238-x

**Published:** 2022-09-09

**Authors:** Chanda Maurya, T. Muhammad, Preeti Dhillon, Priya Maurya

**Affiliations:** 1 Department of Survey Research and Data Analytics, International Institute for Population Sciences, Mumbai, Maharashtra 400088 India; 2 Department of Family & Generations, International Institute for Population Sciences, Mumbai, Maharashtra 400088 India; 3 Department of Population and Development, International Institute for Population Sciences, Mumbai, Maharashtra 400088 India

**Keywords:** Adolescents, Cyberbullying, Depressive symptoms, Longitudinal study, Suicidal ideation

## Abstract

**Background:**

Cyberbullying victimisation is considered a global public health issue concerning the psychological development of adolescents that oftentimes persists into adulthood. The current study explored the longitudinal relationship between cyberbullying victimisation and depression and suicidal ideation among adolescents and young adults, given the scarcity of such studies in poor-resource settings like India.

**Methods:**

Data were drawn from the “Understanding the Lives of Adolescents and Young Adults” (UDAYA- 2015-16 and 2018–19) surveys conducted in two most-populated Indian states of Uttar Pradesh and Bihar. Bivariate and logistic regression analysis was conducted to fulfil the objectives of the study using a sample of 4428 and 11,864 adolescent (aged 10–19 years) male and female cohorts, respectively.

**Results:**

The prevalence of cyberbullying victimization increased from 3.8% to 6.4% among female respondents and 1.9% to 5.6% among male respondents over three years. About 33% of females and 16.6% of males had depressive symptoms in their young adulthood. Nearly 7.5% females compared to 2.3% of males, reported that they have seriously considered attempting suicide in the past one year. Adolscents who experienced cyberbullying victimization were 2.07 times more likely to have depressive symptoms comapared to those who did not experience cyberbullying victimization. Similarly, adolescents who experienced cyberbullying victimization were 2.50 times more likely to have suicidal ideation than their counterparts with no experience of cyberbullying victimization.

**Conclusion:**

The findings suggest that cyberbullying victims are at higher risk of depressive symptoms and suicidal thoughts and these adverse effects persist for longer period. Therefore, cyberbullying and related mental health problems need to be addressed with more efficient strategies such as increased awareness of nuances of online harassments among adolescent and young adult population.

## Introduction

Technological advancements in the last few years have led to increased social interactions that have negative consequences, known as cyberbullying [1]. Being bullied in adolescence is considered a global public health issue concerning the psychological development of adolescents that oftentimes persists into adulthood [2, 3]. Features of cyberbullying such as publicity, permanence (i.e., single acts leading repeated harassments through views and distribution by others) and permeability of online messaging exacerbate the negative effects on adolescents’ mental health [1, 4]. It is documented in multiple studies that adolescents who are cyberbullied by their friends or peers are at greater risk for mental problems including lower levels of self-esteem, feeling of loneliness, depression and suicidal ideation [5–7].

Importantly, victims of cyberbullying among adolescents even if the victimization is at low levels, are at higher risk of future mental health problems [7]. Further, several cross-sectional as well as longitudinal studies have reported that cyberbullying victimization is associated with an increased risk of suicidal ideation, self-harm and suicide attempts [8–12]. Extant literature has examined various factors such as lack of peer support, emotional intelligence, violent behaviour, substance use and access to social media and internet that may influence the association between cyberbullying victimisation and diverse forms of mental health disturbances [1, 13–17]. Also, in multiple studies, adolescent girls’ mental health was more compromised than of boys due to exposure to cyberbullying [18, 19]. The increased prevalence of cyberbullying victimization among female adolescents than males that lead to mental problems and suicidal ideation is reported in multiple studies [11, 20].

A growing body of literature suggests that depressive symptoms and suicidal thoughts are elevated among adolescents and young adults who consume alcohol or smoke tobacco or consume illegal drugs [21–24]. Besides, earlier research has shown the co-occurrence of conduct problems and depressed mood among individuals that increases their risk of suicidal ideation [25]. Furthermore, family socioeconomic status, measured through family income and parental educational attainment, was significantly associated with adolescent mental health [26, 27]. A review of 55 studies among children and adolescents aged four to 18 years revealed that low socioeconomic status was longitudinally related to higher rates of mental health problems. Also,children and adolescents, who were socioeconomically disadvantaged, were two to three times more likely to develop depression and other mental health problems [28]. A list of social determinants, namely, place of residence, religion, race or ethnicity, work and education is also considered to influence depression and suicidal thoughts among adolescents and young adults, especially in particular Indian context [24, 29, 30].

**Fig. 1:**
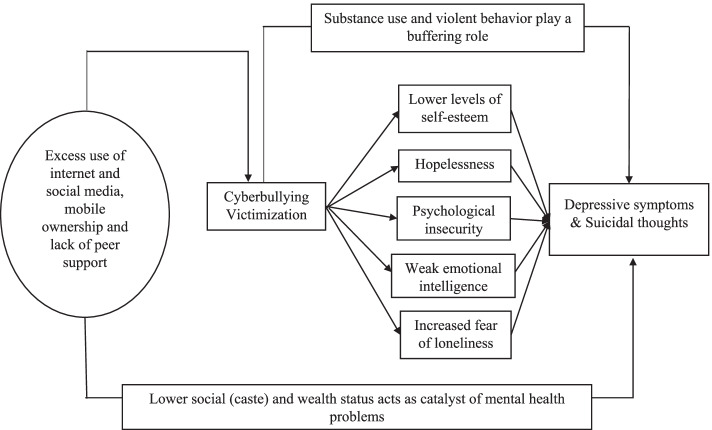
Conceptual framework for depression and sucicial thoughts

Several studies have examined the mechanisms underlying the association between cyberbullying victimization and depression among adolescents and young adults, and found that hopelessness [31], lowered self-esteem [32], psychological insecurity [33] and increased fears of loneliness [34] mediate such association. Similarly, emotional intelligence [35] and depressive symptoms [36] were shown to play a buffering role in the relationship between cyberbullying victimization and suicidal thoughts. Although internet and smartphone use come up with numerous benefits, it also has negative consequences and public health concerns in the present time. Since the use of technologies has increased in terms of internet communities and social networking web sites, chat rooms, and mobile phones, the cyberbullying may occur more frequently with greater repercussions than traditional bullying [37, 38]. Figure 1 demonstrates how cyberbullying victimization can lead to low level of self-esteem, psychological insecurity and fear of lonliness resulting in depression and sucidial ideation. Examining such association can help inform policymakers the importance of interventions in current mental health policy and pre-existing multi-level approaches supporting mental health and anti-bullying efforts.

Adolescents with depression in low and middle-income countries (LMICs) including India carry a double burden of depression and increased risk of engaging in risky behaviors and may impose a health burden on society as a whole [39]. Thus, the aim of this study was to explore the longitudinal relationship between cyberbullying victimisation and depression and suicidal ideation in adolescents and young adults, given the scarcity of studies in poor-resource settings like India. The study also explores such associations after controlling for several confounding variables such as violent behaviour, substance use and peer connection among adolescents and young adults.

## Methods

### Study degisn and setting

The present study used data from the “Understanding the lives of adolescents and young adults” (UDAYA) survey, which was conducted in two most populated Indian states of Uttar Pradesh and Bihar by Population Council under the guidance of Ministry of Health and Family Welfare, Government of India [40]. The first wave was conducted in 2015–2016, and the follow-up survey was conducted after 3 years in 2018–2019. With the use of a multi-stage sampling procedure, the survey gathered information on family, media, community environment, and quality of transitions to young adulthood indicators, and provide the estimates for states as a whole as well as rural and urban of both states. The detail sampling were presented somewhere else [40]. The sample size for Uttar Pradesh and Bihar was 10,161 and 10,433 adolescents aged 10–19 years, respectively.

In wave-1 (2015–16), 20,594 adolescents were interviewed using the structured questionnaire with a response rate of 92%. Moreover, in wave-2 (2018–19), the study again interviewed the participants who were successfully interviewed in 2015–16 and consented to be re-interviewed [40]. Of the 20,594 eligible for the re-interview, the survey reinterviewed 4567 unmarried males and 12,251 females. After excluding the respondents who gave an inconsistent response to age and education in the follow-up survey (3%), the fnal follow-up sample covered 4428 males and 11,864 females, with a follow-up rate of 74% for males and 81% for females [40]. For this study, a total sample of 16,292 adolescent males and females aged 13–23 in 2018–19 considered with participants who were successfully interviewed in 2015–2016 and consented to be re-interviewed.

### Outcome variable

This analysis contains depressive symptoms at wave 2 and suicidal ideation at wave 2 as the outcome variables. Depressive symptoms were assessed using the patient health questionnaire (PHQ-9) [41]; the respondent were asked about the symptoms in the past two weeks. The nine questions included, (a) had trouble falling asleep or sleeping too much, (b) feeling tired or having little energy, (c) poor appetite or eating too much, (d) trouble concentrating on things, (e) had little interest or pleasure in doing things, (f) feeling down, depressed, or hopeless, (g) feeling bad about yourself, (h) been moving or speaking slowly, (i) had thoughts that respondent would be better off dead. All the above questions were asked on a scale of four, i.e., 0 “not at all,” 1 “less than once in a week,” 2 “one week or more” and 3 “nearly every day.” The scale of 27 points was obtained using the *egen* command in STATA 14. The variable was recorded as 1 “yes” meaning meeting criteria for a depressive disorder if the respondent scored five and above on 0–27 scale and 0 “no depressive symptom” if the respondent scored less than five on the scale (0–4, minimal) [41, 42]. Suicidal Ideation at wave 2 was recoded as 1 “yes” if the respondent had seriously considered attempting suicide in the last 1 years and 0 “no” if there was no such attempt [43].

### Key explanatory variable

The main explanatory variable was cyberbullying victimization at wave 1 and wave 2, Cyberbullying victimization was assessed using the questions “Has anyone ever used a cell phone or text messaging to bother or harass you or to spread mean words or pictures about you?” and “Has anyone ever used the internet to bother or harass you or to spread mean words or pictures about you?” and it was categorized as 1 “yes” if the respondent had an affirmative answer to either of the questions and 0 “no” if the respondent did not experience cyberbullying through mobile/internet [44].

### Other explanatory variables

Several potential confounders were selected and included in the current analyses according to the existing literature. Substance use was assessed using the questions “Have you ever had alcohol?” and “Have you ever consumed tobacco products, eg., smoke cigarette, eat paan, gutka etc.?”. The variable was recoded as 1 “yes” if the respondent ever consumed tobacco or alcohol and otherwise “no”. Violent behavior was assessed using the question “These days, we hear a lot about girls/boys getting involved in fights. In the last 12 months, have you been involved in a physical fight with other girls/boys, I mean, beating up other girls/boys, pulling hair, slapping etc?”. It was recoded as 1 “yes” if the respondent had an affirmative answer and otherwise “no” [40].

Age was grouped into 13–17 year, 18–20 years and 21–23 years. Sex was categorized as males and females. Marital status was categorized as single and married. Educational level was categorized as illiterate, up to 8 years and 9 years or above. Ownership of mobile-phone was categorized as “Own mobile”, if the respondent owned mobile phone, “Access family member’s mobile phone” if the respondent can access family member’s mobile phone and otherwise “no” if the respondent had not used mobile phone. Internet access was categorized as “yes” and “no”. Use of social media was categorized as “yes” and “no”. Peer connection was categorized as “good” having 5 or more friends and “bad” having four and less friends. Work status was categorized as “yes” doing paid work in the last 12 months and “no” not doing paid work in the last 12 months. Religion was categorized as Hindu and Non-Hindu. Caste was categorized as schedule caste/schedule tribe (SC/ST), other background classes (OBC) and others. The SC/ST group consists of socioeconomically disadvantaged populations, OBC refers to intermediate group of populations and others consist of people with comparatively higher socioeconomic status.

The survey measured household economic status, using a wealth index composed of household asset data on ownership of selected durable goods, including means of transportation, as well as data on access to a number of amenities. The wealth index was constructed by allocating the scores to a household’s reported assets or amenities. Index scores so constructed ranged from 0 to 57. Households were then ranked according to the index score. This ranked sample was divided into quintiles—that is, five groups, each containing an equal number of households—with the first quintile representing households of the lowest (poorest) wealth status and the fifth quintile representing households with the highest (wealthiest) status [40]. Place of residence was categorized as rural and urban. States were categorized as Uttar Pradesh and Bihar. All the explanatory variables were taken from the follow-up data (wave 2), except the socio-demographic characteristics that did not change over time.

### Statistical analysis

Descriptive and bivariate analysis was used to find out the preliminary results. Further, logistic regression was carried out to examine the association between individual, behavioural and physiological variables at wave 1, with respect to depressive symptoms and suicidal thoughts at wave 2. The multivariable models were assembled as follow: in model 1, key explanatory variables were entered simultaneously to investigate their mutual independence, and model 2 was adjusted with both key explanatory variables and other variables. The model is usually put into a more compact form as follows:

$$\ln\ \left(\frac{Pi}{1- Pi}\right)=\beta 0+\beta 1x1+\dots {\beta}_M{x}_{m-1}$$

Where, *β*0*..., βM* are regression coefficients indicating the relative effect of a particular explanatory variable on the outcome. These coefficients change as per the context in the analysis in the study. The result were presented in the form of odds ratio (OR) with 95% confidence interval (CI).

## Result

Mean age of the male participants was 17.79 years, and mean age of the female participants was 19.43 years in this study. Table 1 shows the socio demographic characteristics of the adolescents and young adults. Nearly half of the respondents were from age-group 18–20 years. Majority of the male respondents (94.08%) were unmarried whereas, more than half of the female respondents (50.79%) were married. Two-third of male respondents had their own mobile phone while, same figure for females were only 36.98%. Similarly, the prevalence of internet access and social media use were higher among male respondents (73.92 and 62.47%) than female respondents (33.64 and 22.95%). Male respondents had a better peer connection than female respondents. The prevalence of work participation among male respondents was 44.65% and among female respondents, it was 21.95%. Majority of the respondents were rural residents in this study.

**Table 1:** Socio- demographic charactisitcs of the study population

Background characteristics	Males		Females	
	Frequency	Percent	Frequency	Percent
**Substance use**				
No	3050	68.87	11,543	95.54
Yes	1378	31.13	539	4.46
**Violent behaviour**				
No	3116	70.37	11,309	93.61
Yes	1312	29.63	772	6.39
**Age (in years)**				
13–17	1423	32.13	1135	9.39
18–20	2195	49.58	5958	49.32
21–23	810	18.29	4989	41.29
**Marital status**				
Married	262	5.92	6136	50.79
Single	4166	94.08	5945	49.21
**Educational level**				
Illiterate	104	2.34	1482	12.27
Up to 8 years	1572	35.5	3944	32.64
9 and more years	2752	62.15	6656	55.09
**Mobile phone access**				
No	156	3.52	385	3.19
Own mobile phone	3010	67.97	4468	36.98
Use family member’s mobile phone	1262	28.51	7228	59.83
**Internet access**				
No	1155	26.08	8017	66.36
yes	3273	73.92	4064	33.64
**Use of social media**				
No	1662	37.53	9308	77.05
yes	2766	62.47	2773	22.95
**Peer connection**				
Bad	2142	48.38	8435	69.82
Good	2286	51.62	3646	30.18
**Work**				
Yes	1977	44.65	2651	21.95
No	2451	55.35	9430	78.05
**Religion**				
Hindu	3761	84.93	9548	79.03
Non-Hindu	667	15.07	2533	20.97
**Caste**				
SC/ST	1210	27.34	3159	26.15
OBC	2424	54.74	6665	55.17
Others	794	17.93	2257	18.68
**Wealth index**				
Poorest	503	11.37	1542	12.77
Poorer	883	19.94	2237	18.51
Middle	986	22.26	2629	21.76
Richer	1053	23.78	3014	24.95
Richest	1003	22.66	2658	22
**Place of residence**				
Urban	743	16.78	1879	15.55
Rural	3685	83.22	10,203	84.45
**State**				
Uttar Pradesh	3008	67.93	8408	69.6
Bihar	1420	32.07	3673	30.4
**Total**	**4428**		**12,081**	

**Fig. 2:**
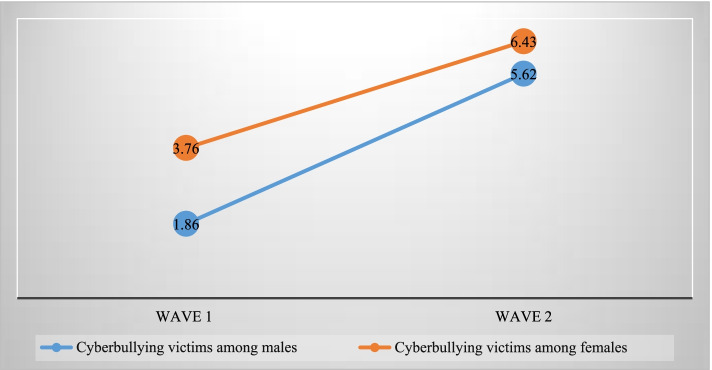
Trend of cyberbullying victimization through mobile/internet among adolescent males and females during wave-1 to wave-2 (2015–2016 to 2017–2018)

**Fig. 3:**
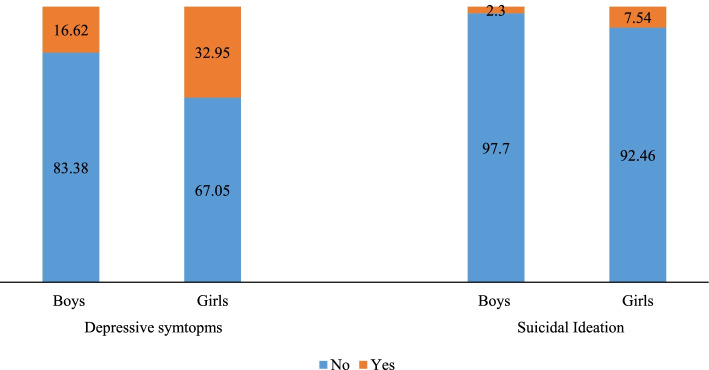
Percentage of males and females having depressive symptoms and suicidal ideation at wave 2

The prevalence of cyberbullying victimization has increased from 3.76 and 1.86% to 6.43 and 5.62% among male and female respondents between wave-1 to wave-2, respectively (Fig. 2). About 16.62% male respondents and 32.95% female respondents had depressive symptoms. Further, the prevalence of suicidal ideation was higher among females than males. About 7.54% of females seriously considered attempting suicide in the past 1 year while the same figure for male respondents was 2.3% (Fig. 3).

Table 2 shows the results from the bivariate association for depressive symptoms and suicidal ideation with different socio-demographic variables for male and females separately. Adolescents who experienced cyberbullying at wave 1 had higher prevalence of depressive symptoms (males: 25.8% and females: 41.5%) and suicide ideation (males: 6.2% and females: 11%) at wave 2. Similarily, adolescents who experienced cyberbullying at wave 2 had higher prevalence of depressive symptoms (males: 27.8% and females: 49.1%) and higher suicidal ideations (males: 7.8% and females: 12.1%). It was found that depressive symptoms were significantly more prevalent among females who were ever cyberbullied at both waves. Adoelscents with depressive symptoms at wave 1 had higher prevalence of depressive symtopms (males: 12.1% and females: 49.1%) and suicidal ideations (males: 7.8% and females: 12.1%) at wave-2.

**Table 2:** Bivariate association of depressive symptoms and suicidal ideation among adolescents (at wave-2) with cyberbullying victimization and other background characteristics

Background characteristics	Depressive symptoms at wave 2				Suicidal ideation at wave 2			
	Males		Females		Males		Females	
	Percent	Chi square *p*-value	Percent	Chi square *p*-value	Percent	Chi square *p*-value	Percent	Chi square *p*-value
**Cyberbullying victimization at wave1**		0.012		< 0.001		0.045		0.011
No	16.2		34.4		2.8		8.1	
Yes	25.8		41.5		6.2		11	
**Cyberbullying victimization at wave 2**		< 0.001		< 0.001		< 0.001		< 0.001
No	15.4		33		2.5		7.4	
Yes	33.2		53.2		7.9		17.8	
**Depressive symptoms at wave 1**		< 0.001		< 0.001		< 0.001		< 0.001
No	15.5		31.4		2.4		7.4	
Yes	27.8		49.1		7.8		12.1	
**Substance use**		< 0.001		< 0.001		< 0.001		< 0.001
No	14.53		34.22		2.05		8.07	
Yes	20.78		48.14		4.66		13.13	
**Violent behaviour**		< 0.001		< 0.001		< 0.001		0.004
No	14.24		34.13		2.04		8.06	
Yes	21.03		43.17		4.53		10.91	
**Age group**		< 0.001		< 0.001		< 0.001		< 0.001
13–17	10.76		21.62		1.25		4.39	
18–20	18.87		34.23		3.93		8.45	
21–23	20		39.01		2.79		9.03	
**Marital status**		0.238		< 0.001		0.178		< 0.001
Married	19.42		41.81		4.37		10.48	
Single	16.3		27.99		2.77		6.13	
**Educational level**		< 0.001		< 0.001		< 0.001		0.007
Illiterate	1.8		4.91		15.32		38.1	
Up to 8 years	4.02		6.1		12.19		34.86	
9 or more years	6.79		10.82		18.79		33.92	
**Ownership of mobile**		< 0.001		< 0.001		< 0.001		< 0.001
**Phone**								
No	14.93		32.57		2.99		5.26	
Own mobile phone	19.06		40.05		3.49		9.88	
Use family member’s mobile phone	10.08		31.21		1.22		7.28	
**Internet access**		< 0.001		0.036		0.023		0.436
No	9.67		33.99		1.74		8.43	
Yes	18.22		35.85		3.14		8.03	
**Use of social media **		< 0.001		0.245		0.023		0.883
No	10.27		34.43		2.01		8.28	
Yes	19.28		35.55		3.23		8.2	
**Peer connection**		0.021		0.007		0.012		0.228
Bad	14.95		33.95		2.12		8.05	
Good	17.55		36.5		3.38		8.71	
**Work**		< 0.001		0.006		< 0.001		0.003
Yes	20.25		37.07		4.0		9.72	
No	13.64		34.14		2.0		7.87	
**Religion**		0.918		0.682		0.225		< 0.001
Hindu	16.47		34.67		2.98		8.77	
Non-Hindu	16.31		35.1		2.15		6.4	
**Caste**		0.571		< 0.001		0.036		< 0.001
SC/ST	16.57		37.31		3.96		9.99	
OBC	16.01		34.87		2.55		8.44	
Other	17.56		30.85		2.27		5.26	
**Wealth index**		0.378		< 0.001		0.894		< 0.001
Poorest	14.01		37.25		2.9		10.72	
Poorer	15.59		36.11		2.43		9.38	
Middle	15.64		35.66		2.75		8.92	
Richer	17.41		35.43		2.76		8.23	
Richest	17.41		31		3.22		5.68	
**Place of residence**		0.515		0.015		0.024		0.129
Urban	16.84		36.02		3.47		8.72	
Rural	16.11		33.87		2.34		7.94	
**State**		0.19		< 0.001		0.793		< 0.001
Uttar Pradesh	15.74		30.32		2.78		7.22	
Bihar	17.2		38.63		2.91		9.17	
**Total**	**16.44**		**34.76**		**2.85**		**8.26**	

The estimates from the logistic regression analysis for depressive symptoms among adolescents are presented in Table 3. Adolscents who experienced cyberbullying victimization at wave1 and wave-2 were 1.58 and 2.45 times more likely to have depressive symptoms at wave 2, comapared to those who did not experience cyberbullying victimization (Model-1). After adujsuting for socio-demographic variables, the magnitude of odds had slightly changed. Late adolescents aged 18–20 and 21–23 years had significantly higher odds of depressive symptoms as compared to younger adolescents (those aged 13–17 years) (Model-2).

**Table 3:** Logistic regression estimate for depressive symptoms by background characteristics among adolescents at wave-2

Background Characteristics	Model-1: OR (CI)	Model-2: AOR (CI)
**Cyberbullying victimization at wave 1**		
No®		
Yes	1.15*(0.98 1.35)	0.97 (0.82 1.14)
**Cyberbullying victimization at wave 2**		
No®		
Yes	2.45***(2.17 2.75)	2.07***(1.83 2.34)
**Depression at wave 1**		
No®		
Yes	2.14***(1.95 2.34)	1.77***(1.61 1.94)
**Substance use**		
No®		
Yes	1.76***(1.48 2.09)	1.56***(1.31 1.86)
**Suicidal ideation**		
No®		
Yes	0.97 (0.88 1.08)	1.64***(1.46 1.84)
**Violent behaviour**		
No®		
Yes	0.90*(0.8 1.01)	1.3***(1.14 1.48)
**Age group (in years)**		
13–17®		
18–20		1.47***(1.29 1.69)
21–23		1.56***(1.35 1.82)
**Sex**		
Male®		
Female		2.64***(2.33 2.98)
**Marital status**		
Single		
Married®		1.65***(1.50 1.82)
**Educational level**		
Illiterate®		
Up to 8 years		1.06 (0.93 1.2)
9 or more years		1.08 (0.95 1.23)
**Ownership of mobile phone**		
No®		
Own mobile		0.87 (0.68 1.09)
Access family member’s mobile phone		0.79**(0.63 0.99)
**Internet access**		
No®		
Yes		1.09 (0.97 1.23)
**Use of social media **		
No®		
Yes		1.03 (0.91 1.16)
**Peer connection**		
Bad®		
Good		1.17***(1.08 1.26)
**Work**		
No®		
Yes		0.83***(0.76 0.90)
**Religion**		
Hindu®		
Non-Hindu		1.16***(1.05 1.27)
**Caste**		
SC/ST		1.11 (0.98 1.25)
OBC		1.02 (0.92 1.13)
Other®		
**Wealth index**		
Poorest		1.15*(1 1.34)
Poorer		1.16**(1.02 1.32)
Middle		1.12*(0.99 1.26)
Richer		1.11**(1 1.23)
Richest®		
**Place of residence**		
Urban®		
Rural		0.92**(0.85 0.99)
**State**		
Uttar Pradesh®		
Bihar		1.24***(1.14 1.33)
**Constant**	0.34***(0.32–0.35)	0.08***(0.06 0.11)

® Reference category, *AOR* Adjusted odds ratio, *CI* Confidence interval, *OBC* Other backward class, *OR* Odds ratio, *SC/ST* Schedule Caste/Schedule Tribes

^*^p < 0.1

^**^p < 0.05

^***^p < 0.01

The estimates from the logistic regression analysis for suicidal ideation among adolescents are presented in Table 4. Adolscents who experienced cybullying victimization at wave 2, depression at wave 1 and suicidal ideation at wave 1, were 2.87, 1.74 and 2.25 times more likely to have suicidal ideation at wave 2 than their counterparts (Model-1). After controlling for socio-demographic characteristics, the magnitude of odds had changed slightly. Those who used substance or had violent behavior at wave-2 were more likely to have suicidal ideation. Adolescents aged 18–20 (OR: 1.80; *p* = < 0.01, CI: 1.36–2.38) and 21–23 years (*p* = < 0.01, CI: 1.19–2.18) were significantly more likely to have suicidal ideation as compared to their younger (aged 13–17 years) counterparts. The likelihood of suicidal ideations were three times higher among females than males (AOR: 3.16; CI: 2.46–4.05) (Model-2).

**Table 4:** Logistic regression estimate for suicidal thoughts by background characteristics among adolescents at wave-2

Background Characteristics	Model-1: OR (CI)	Model-2: AOR (CI)
**Cyberbullying victim at wave 1**		
No®		
Yes	1.14 (0.88–1.47)	1.08 (0.83–1.4)
**Cyberbullying victim at wave 2**		
No®		
Yes	2.87***(2.43–3.4)	2.50***(2.09–2.98)
**Depression at wave 1**		
No®		
Yes	1.74***(1.49–2.02)	1.46***(1.25–1.7)
**Suicidal thoughts at wave 1**		
No®		
Yes	2.25***(1.78–2.85)	1.92***(1.51–2.45)
**Substance use**		
No®		
Yes	1 (0.82–1.22)	1.47***(1.17–1.85)
**Violent behaviour**		
No®		
Yes	0.98 (0.82–1.17)	1.58***(1.29–1.92)
**Age group (in years)**		
13–17®		
18–20		1.80***(1.36–2.38)
21–23		1.61***(1.19–2.18)
**Sex**		
Male®		
Female		3.16***(2.46–4.05)
**Marital status**		
Single		
Married®		1.5***(1.26–1.79)
**Educational level**		
Illiterate®		
Up to 8 years		0.93 (0.76–1.14)
9 or more years		0.79**(0.64–0.98)
**Ownership of mobile phone**		
No®		
Own mobile		1.31 (0.82–2.11)
Use family member’s mobile phone		1.19 (0.75–1.9)
**Internet access**		
No®		
Yes		0.95 (0.76–1.17)
**Use of social media **		
No®		
Yes		1.12 (0.9–1.41)
**Peer connection**		
Bad®		
Good		1.19**(1.04–1.36)
**Work**		
No®		
Yes		1.26***(1.09–1.47)
**Religion**		
Hindu®		
Non-Hindu		0.77***(0.64–0.92)
**Caste**		
SC/ST		1.42***(1.13–1.79)
OBC		1.35***(1.1–1.66)
Other®		
**Wealth index**		
Poorest		1.58***(1.22–2.04)
Poorer		1.46***(1.15–1.85)
Middle		1.4***(1.13–1.74)
Richer		1.28**(1.05–1.56)
Richest®		
**Place of residence**		
Urban®		
Rural		0.75***(0.66–0.87)
**State**		
Uttar Pradesh®		
Bihar		0.98 (0.85–1.12)
**Constant**	0.05***(0.05–0.06)	0.01***(0–0.01)

^*^p < 0.1

^**^p < 0.05

^***^p < 0.01

® Reference category, *AOR* Adjusted odds ratio, *CI* Confidence interval, *OBC* Other backward class, *OR* Odds ratio, *SC/ST* Schedule Caste/Schedule Tribes

## Discussion

Cyberbullying victimization has become a global phenomenon in a digitalized world, predominantly addressed in the developed world. In an attempt to address research gaps in understanding cyberbullying in India, this is the first study to elucidate the longitudinal association between cyberbullying victimization and mental health among Indian adolescents and young adults. With the rapid advancement and revolutionization of information and communication technologies (ICTs), adolescents and young adults use smartphone devices, internet and social networking services more frequently, which escalates the issue of cyberbullying abruptly, resulting in psychiatric problems and negative thoughts among victims [37, 45, 46]. The present study observed a strong association of cyberbullying victimization with depression and suicidal thoughts among adolescents and young adults. The relationships between cyberbullying victimization with depression symptoms and suicidal thoughts were stronger during the cross-sectional period in this study after adjusting for baseline characteristics.

The overall prevalence of cyberbullying victimization through mobile or internet has increased over the study period. Similarly, a repeated study from the United Nations observed an increasing trend of prevalence of cyberbullying victimization from 6 to 11% between the years, 2005 and 2010 [47]. This increase indicates that more number of adolescents have access to digital means of communication nowadays than in the past [48]. However, the literature is not uniform on this issue across developed and developing countries. The prevalence of cyberbullying victimization was lower in the present study than reported in other studies from India [49–51]. A clinical study from Turkey found that 62.2% of adolescents were victims of cyberbullying, which was much higher than non-clinical adolescent samples [52]. The possible explanation of the differences in prevalence in our study and other studies could be due to changes in methodology, survey design and access to and prevalence of internet use. Consistent with previous evidence from India [43, 53], this study also found that a considerable number of adolescents and young adults had depressive symptoms and suicidal thoughts, which was higher among adolescent and young adult females than males.

Our findings are in line with previous literature that suggested that victims of cyberbullying were more likely to be females [19, 45, 54, 55]. Mesch (2009) found in their study that females were two times more likely to be victims of cyberbullying at least once than males [54]. This gender pattern may be delineated by cultural stereotypes where male respondents try to tackle the problem by themselves and avoid reporting of victimization as doing so might foul up their sense of masculinity. In reverse, females might encourage reporting of victimization as they perceive cyberbullying as serious problem and give more importance on peer relationships as well [45, 55]. However, few studies reported male respondents were more likely to be victims of cyberbullying [7, 8] and another study found no significant gender differences in victimization [56]. Gender-specific studies are warranted on this direction.

Furthermore, our findings are in concordance with prior studies that have shown that cyberbullying victimization has a negative impact on mental health [7, 14, 18] and suicidal thoughts [57–59]. This association was more prominent among those who experienced cyberbullies in follow-up period of the study. Fahy et al. (2016) also found in their longitudinal study that baseline cyberbullying victimization predicted depressive symptoms at follow-up period [7]. Similarly, another longitudinal study reported that cyberbullying victimization predicted negative thoughts and suicidal ideation among adolescents [56]. Researchers also coined this phenomenon as ‘Cyberbullicide’, which is defined the act of suicide influenced by experiences with online aggression [60]. Previous evidence has shown that being cyberbullied increased the chances of developing pessimistic slant such as loneliness, feeling dehumanized and helpless, leading to greater risk for depression and suicidal thoughts [37, 45, 55, 61]. A shred of literature also stated that the relationship between cyberbullying victimization and mental illness generates a vicious cycle. Adolescents with depressive symptoms or poor mental health may be more likely to engage in social media and the internet to divert or covert from their emotions. Consequently, they become more exposed and vulnerable to victimization of cyberbullying, which leads to more risk of severe depression and suicidal thoughts [8, 37, 59, 61]. Some authors also argued that these incidents take place in a bidirectional way, such as either depressive symptoms can be a cause or consequence of cyberbullying or vice versa [37, 48, 62].

Beside cyber victimization, having depression at baseline, substance uses and having violent bahviour were important risk factors for depression and suicidal thoughts after adjusting for other covariates. This finding suggests that adolescents having depressive symptoms may be more prone to substance use when facing negative experiences such as cyberbullying victimization. It may also reflect that adolescents suffering from online attacks were at higher risk of using substances to cope with the situations [55, 57]. We found that violent behavior and depression at baseline were closely related with depression and negative thoughts among adolescents as they are interconnected. Violent behaviour often coexists with anxiety and adolescence is a period in which people often feel disgruntled, resentful or irritable. These kinds of emotions lead to violent behavior among adolescents resulting in depression and negative thoughts [63, 64].

The present study revealed variation in prevalence of depression and suicidal ideation across different socio-demographic characteristics. Older adolescents and female participants were more likely to have depression and suicidal thoughts than their younger and male counterparts. This finding is partialy explained by biological theory and peer events occurring around the older and girl adolescents. Additionally, adolescent females may develop more negative perceptions of themselves in certain domains [21, 65]. Findings also indicate that being single was a protective factor for depressive symptoms and suicidal thoughts. This can be partially explained by the fact that married girls could not express their opinion compared to unmarried respondents [43]. This study revealed that having more number of friends leads to poor mental health and suicidal ideation among adolescents and the reasons might be feeling of insecurity, adverse life events among peer group [65]. However, having a good peer connection and social connctedness should be further investigated in light of their impact on adolescents’ and young adults’ mental health status.

This study is not without limitations. Although we utilized longitudinal data for the analysis, all measures included in the current study were self-reported which may lead to recall and desirability bias, especially in case of more sensitive questions. For example, some respondents may not have disclosed their experiences of cyberbullying victimization. Further, information on victimization of cyberbullying was derived from a single question at the time of the survey, but the severity of experience has not been considered in this study. In addition, the study utilized data from the year 2015–2016 and 2018–2019, so interpretation of the findings could not measure recent ongoing situations such as impact of the COVID-19 pandemic when online activity has increased. Therefore, such aforesaid conditions come up with potentially increased cyberbullying victimization rates among adolescents and young adults which need to be further investigated.

## Conclusion

The present study adds to the growing body of evidence on the impact of cyberbullying victimization on depressive symptoms and suicidal ideation among adolescents and young adults. Our findings suggest that cyberbullying victims are at higher risk of depression and suicidal ideation. Therefore, cyberbullying and related mental health problems need to be addressed with more efficient strategies such as increased awareness of nuances of online harassments among adolescent and young adult population. Also, emphasis should be given to promoting healthy internet use, safeguarding online activities, and providing knowledge of coping and help-seeking skills. Mental health is considered as a major public health concern in our country in National Mental Health Policy 2014. In spite of that, mental health problems associated with cyberbullying among adolescents and young adults should be considered specifically in Rashtriya Kishore Swasthya Karyakram as this program targets adolescents and young adults. Additionally, the policy planning approach should be focused on prevention strategies in case of cyberbullying victimization. A multi-level approach at the individual, household, community, state and national level can be adapted to provide a safer online platform for adolescents and young adults in the country.

## Data Availability

Data for the study were extracted from the two waves of the “Understanding the Lives of Adolescents and Young Adults (UDAYA) survey” conducted by Population Council. The data is available only on request from: https://dataverse.harvard.edu/dataset.xhtml?persistentId=doi:10.7910/DVN/RRXQNT https://dataverse.harvard.edu/dataset.xhtml?persistentId=doi:10.7910/DVN/ZJPKW5
